# Prediction model of acute kidney injury induced by cisplatin in older adults using a machine learning algorithm

**DOI:** 10.1371/journal.pone.0262021

**Published:** 2022-01-18

**Authors:** Takaya Okawa, Tomohiro Mizuno, Shogo Hanabusa, Takeshi Ikeda, Fumihiro Mizokami, Takenao Koseki, Kazuo Takahashi, Yukio Yuzawa, Naotake Tsuboi, Shigeki Yamada, Yoshitaka Kameya

**Affiliations:** 1 Department of Clinical Pharmacy, Fujita Health University School of Medicine, Toyoake, Japan; 2 Department of Information Engineering, Meijo University, Nagoya, Japan; 3 Department of Pharmacy, National Center for Geriatrics and Gerontology, Obu, Japan; 4 Department of Biomedical Molecular Sciences, Fujita Health University School of Medicine, Toyoake, Japan; 5 Department of Nephrology, Fujita Health University School of Medicine, Toyoake, Japan; National Institutes of Health, UNITED STATES

## Abstract

**Background:**

Early detection and prediction of cisplatin-induced acute kidney injury (Cis-AKI) are essential for the management of patients on chemotherapy with cisplatin. This study aimed to evaluate the performance of a prediction model for Cis-AKI.

**Methods:**

Japanese patients, who received cisplatin as the first-line chemotherapy at Fujita Health University Hospital, were enrolled in the study. The main metrics for evaluating the machine learning model were the area under the curve (AUC), accuracy, precision, recall, and F-measure. In addition, the rank of contribution as a predictive factor of Cis-AKI was determined by machine learning.

**Results:**

A total of 1,014 and 226 patients were assigned to the development and validation data groups, respectively. The current prediction model showed the highest performance in patients 65 years old and above (AUC: 0.78, accuracy: 0.77, precision: 0.38, recall: 0.70, F-measure: 0.49). The maximum daily cisplatin dose and serum albumin levels contributed the most to the prediction of Cis-AKI.

**Conclusion:**

Our prediction model for Cis-AKI performed effectively in older patients.

## Introduction

Acute kidney injury (AKI) contributes to resource utilization and higher healthcare costs in the hospital. The monitoring and management of AKI have been reported by Palevsky PM et al. in their study Kidney Disease Outcomes Quality Initiative (KDOQI) US commentary on the 2012 Kidney Disease Improving Global Outcomes (KDIGO) clinical practice guideline for acute kidney injury [1]. Patients with malignancies have an increased risk for AKI due to tubular damage, interstitial nephritis, and thrombotic microangiopathy caused by chemotherapy [2]. In addition, AKI induced by chemotherapy has a poor prognosis [3,4].

Cisplatin-based chemotherapy is the first-line therapy for many solid carcinomas [5,6]. Cisplatin is eliminated via the renal tubules, and therefore, may cause tubular injury, resulting in AKI. To prevent cisplatin-induced AKI (Cis-AKI), aggressive or short-duration hydration with magnesium supplementation [7,8] is recommended. Nevertheless, the incidence of Cis-AKI remains high, and early detection and prediction of Cis-AKI are essential in the management of patients on chemotherapy with cisplatin [9,10].

Computer algorithm systems that automatically track serum creatinine changes have been developed to detect AKI in the early stage [11–13]. However, these alert systems for AKI did not improve survival or reduce the rate of renal replacement therapy [14]. Due to the lag between the increase in serum creatinine and the onset of renal injury [15], these systems may not effectively detect early-stage AKI.

A machine learning algorithm was subsequently developed for accurately detecting AKI [16–19]. Tomašev et al. proposed a recurrent neural network utilizing individual electronic health records, which would process the data one step at a time and build an internal memory [16]. This algorithm system sends an alert when the predicted probability exceeds a specified operating-point threshold. One report has also proposed new machine learning algorithms, specifically for patients with AKI in the intensive care unit [19].

In this study, we intended to evaluate the performance of a prediction model for Cis-AKI. In addition, we investigated the parameter contributing the prediction of AKI in the model.

## Materials and methods

### Data source and study design

Japanese patients, who received cisplatin as first-line chemotherapy at Fujita Health University Hospital from April 2006 to December 2013, were enrolled in the study. Data pertaining to the first course of chemotherapy were recorded. Patients who received one or more courses of chemotherapy prior to the enrollment were excluded from this study. Further, to eliminate the bias related to the increase in toxicity due to angiographic agents and that related to the reduction for renal toxicity, we excluded the patients who received cisplatin via intra-arterial injection.

### Baseline characteristics

The present study used sex, age, baseline body surface area, maximum daily cisplatin dose, baseline serum creatinine, baseline serum albumin, history of diabetes mellitus, and history of cardiovascular events as predictors of Cis-AKI. These predictors were identified as risk factors for Cis-AKI in previous reports [20–22]. The aim of current prediction model was to screen the risk of all patients who received cisplatin based chemotherapy. Therefore, patients treated with different dosages of cisplatin were included in this study. Since serum electrolytes are affected by water intake and dehydration, we did not include this as a predictor in this study. Severity of Cis-AKI was evaluated during the first course chemotherapy; therefore, the maximum daily cisplatin dose was included as a predictor of Cis-AKI. In general, the value of estimated glomerular filtration rate (eGFR) is used for evaluating the renal function. Since the data of eGFR consists of age, gender, and serum creatinine, eGFR was not used as a predictor of Cis-AKI in the present study.

Cis-AKI was graded according to the Common Terminology Criteria for Adverse Events (CTCAE), version 4.0. In the present study, CTCAE grade 1 (more than 1.5 times from the baseline or 0.3 mg/dL increase in serum creatinine) was defined as Cis-AKI.

### Machine learning methods

Machine learning was performed using Prediction One ver 2.1.1.3 (Sony Corporation, Tokyo, Japan). In Prediction One, all predictors were used for building an ensemble model consisting of neural networks and gradient boosting decision trees. Neural networks are able to learn representations of both key factors and their interactions from data [23], and gradient boosting decision trees support decision making, similar to a flowchart estimating the risk for an outcome [24]. Hyperparameters were used to improve the models by selecting the best model. Moreover, Prediction One can identify the rank of contribution as a predictive factor based on sensitivity analysis. Data from April 2006 to December 2012 were used for the development data sets, and data from January 2013 to December 2013 were used as the validation data sets. The data sets were shuffled before machine learning. The main metrics for evaluating the machine learning model were the area under the curve (AUC), accuracy, precision, recall, and F-measure. In addition, the rank of contribution as a predictive factor of Cis-AKI was determined by Prediction One.

### Statistical analysis

Data are displayed as the means ± S.D. To assess the homoscedasticity of the data, we conducted Levene’s test. The Student’s t-test and Welch t-test were used to compare homoscedastic and non-homoscedastic data, respectively. The Chi-square test was used to analyze nominal data. A two-sided *p*-value of < 0.05 was considered significant in all statistical analyses. SPSS version 22.0 (SPSS Inc., Chicago, IL, USA) was used for all statistical analyses.

### Ethics approval

This study was approved by the ethics board of Fujita Health University Hospital (ethics committee approval number: HM20-333). Because it was a retrospective cohort study, an opt-out approach for informed consent was used as approved by the ethics board.

## Results

### Study participants

**Fig 1:**
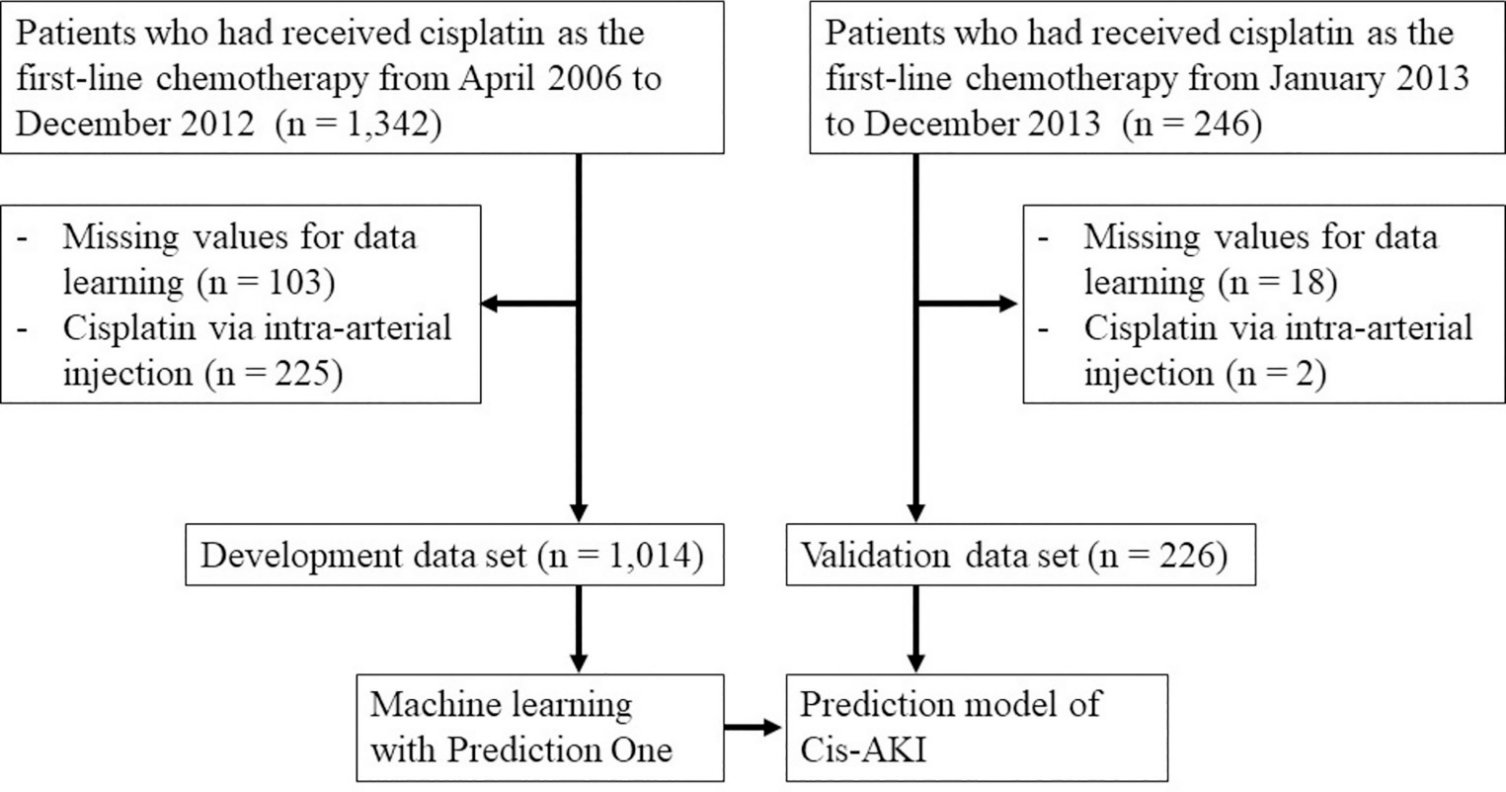
Study protocol. Cis-AKI: Cisplatin-induced acute kidney injury.

A total of 1,014 and 226 patients were assigned to the development and validation data groups, respectively (Fig 1). The characteristics of these data sets are shown in Table 1. The number of patients who were 65 years or older in the development and validation data groups were 490 (39.4%) and 125 (55.3%), respectively. The number of patients, who were 75 years or older in the development and validation data groups were 115 (11.3%) and 31 (13.7%), respectively. The patients were divided into four groups based on age. The serum albumin level and number of patients with history of cardiovascular diseases (CVD) in the development data group were significantly higher than those of the validation data set (serum albumin: *p* = 0.001, history of CVD: *p* = 0.010).

**Table 1:** Baseline characteristics.

Characteristics	Development data	Validation data	*p* value
	(*n* = 1014)	(*n* = 226)	
Sex			
Male (*n*)	725 (69.6%)	162 (71.7%)	0.956
Female (*n*)	289 (30.4%)	64 (28.3%)	
Age, mean (range)	62.5 ± 11.0	63.8 ± 11.6	0.128
≧65 years old (*n*)	490 (39.4%)	125 (55.3%)	0.057
≧75 years old (*n*)	115 (11.3%)	31 (13.7%)	0.316
BSA (m^2^)	1.59 ± 0.18	1.61 ± 0.17	0.108
Maximum daily cisplatin dose (mg)	80.7 ± 32.3	84.0 ± 36.6	0.211
Serum creatinine (mg/dl)	0.75 ± 0.22	0.74 ± 0.21	0.694
Serum albumin (mg/dl)	3.83 ± 0.54	3.68 ± 0.66	0.001
History of DM (*n*)	134 (13.2%)	30 (13.3%)	0.981
History of CVD (*n*)	93 (9.17%)	9 (3.98%)	0.010
Cis-AKI (*n*)	184 (18.1%)	29 (12.8%)	0.055

BSA: Body surface area, DM: Diabetes mellitus, CVD: Cardio vascular disease, Cis-AKI: Cisplatin-induced acute kidney injury.

### Performance for predicting Cis-AKI

**Fig 2:**
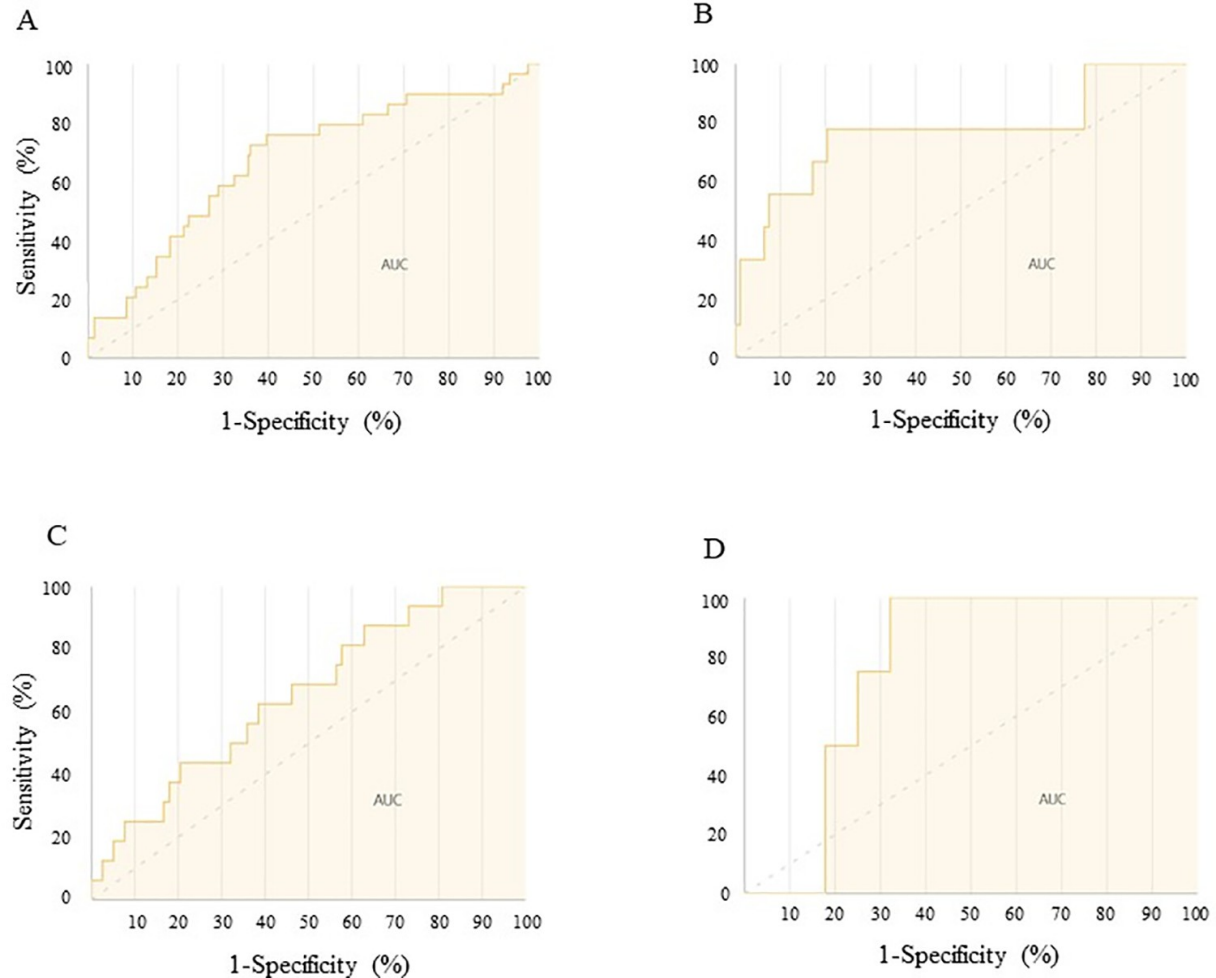
Receiver operating characteristic curve for prediction of the Cis-AKI. A: All patients, B: Under 65 years old, C: 65–74 years old, D: 75 years or older. Cis-AKI: Cisplatin-induced acute kidney injury, AUC: Area under the curve.

The prediction model was established by learning using the development data set (n = 1,014) and evaluated using the validation data set (n = 226). Table 2 shows the result of the model performance test assessed using an independent data set. The model’s performance was evaluated in terms of the AUC, accuracy, precision, recall, and F-measure. The receiver operating characteristic curves are also shown in Fig 2. In patients who were aged 75 years or older, the current prediction model showed the highest performance (AUC: 0.76, accuracy: 0.71, precision: 0.31, recall: 1.00, F-measure: 0.47).

**Table 2:** Model performance for predicting Cis-AKI.

Characteristics	AUC	Accuracy	Precision	Recall	F-measure
All patients	0.67	0.65	0.22	0.69	0.33
< 65 years old	0.77	0.72	0.21	0.78	0.33
65–74 years old	0.65	0.48	0.22	0.81	0.35
≧75 years old	0.76	0.71	0.31	1.00	0.47

Cis-AKI: Cisplatin-induced acute kidney injury, AUC: Area under the curve.

The rank of contribution as a predictive factor of Cis-AKI is listed in Table 3. Serum albumin levels, BSA, and maximum daily cisplatin dose contributed the most to the prediction of Cis-AKI.

**Table 3:** Rank of contribution as a prediction factor of Cis-AKI.

Characteristics	Male sex	Age	BSA	Serum creatinine	Serum albumin	History of DM	History of CVD	Maximum daily cisplatin dose
All patients	8	4	1	5	3	6	7	2
< 65 years old	7	3	5	6	2	8	1	4
65–74 years old	7	4	2	5	1	8	6	3
≧75 years old	6	8	2	5	3	1	7	4
Average	7.0	4.8	2.5	5.3	2.3	5.8	5.3	3.3

Cis-AKI: Cisplatin-induced acute kidney injury, BSA: Body surface area, DM: Diabetes mellitus, CVD: Cardiovascular disease.

The parameter range of the highest contribution as a prediction factor of Cis-AKI in each parameter is shown in Table 4. High dose of cisplatin (100–120 mg/day) and low serum albumin level (1.30–3.10 mg/dL) were the risk factors of Cis-AKI in all patients. In patients who were 65–74 years old, high dose of cisplatin (120–150 mg/day), low serum albumin level (3.00–3.40 mg/dL), and low BSA (1.42–1.48 m^2^) were the risk factors of Cis-AKI. History of CVD contributed to the prediction of Cis-AKI in patients who were under 65 years old. The gender, age, and serum creatinine levels did not contribute the prediction of Cis-AKI in all age groups.

**Table 4:** The parameter range of highest contribution as a prediction factor of Cis-AKI.

Characteristics	All patients	< 65 years old	65–74 years old	≧75 years old
Maximum daily cisplatin dose	100–120 mg/day	75.0–88.0 mg/day	120–150 mg/day	110–130 mg/day
Serum albumin level	1.30–3.10 mg/dL	1.30–3.20 mg/dL	3.00–3.40 mg/dL	3.40–3.60 mg/dL
BSA	1.58–1.63 m^2^	1.86–2.12 m^2^	1.42–1.48 m^2^	1.76–1.96 m^2^
Age	68.0–71.0 year	62.0–64.0 year	68.0–70.0 year	82.0–89.0 year
Serum creatinine	0.87–1.01 mg/dL	0.50–0.56 mg/dL	0.30–0.54 mg/dL	0.97–1.07 mg/dL

Cis-AKI: Cisplatin-induced acute kidney injury, BSA: Body surface area.

## Discussion

In the current study, we constructed a prediction model for Cis-AKI in older adults using a machine learning algorithm. Although cisplatin is a “traditional anti-cancer agent,” it is used in combination chemotherapy with immune checkpoint inhibitors [25,26]. Since Cis-AKI is a risk factor for mortality [3,27] and therapeutic interventions for Cis-AKI are limited, preventing AKI improves the treatment outcome of cisplatin-based chemotherapy. The preventive approaches include hydration and magnesium administration; however, these approaches do not entirely prevent Cis-AKI. In addition, AKI events might be overlooked by the lag between the increase in serum creatinine and the onset of renal injury [15]. The prediction model of Cis-AKI could be more effective than renal function monitoring and traditional preventive approaches in improving the mortality and prognosis of patients with cancer.

Our result suggested that history of CVD contributed less to the prediction of Cis-AKI in older patients than in patients who were under 65 years old. Since the mean age of the subjects was 60 years old in the previous study which identified the history of CVD as a risk factor of severe Cis-AKI [20], our result was consistent with the result of the previous study. CVD is associated with arteriosclerosis and glomerular sclerosis. Therefore, our result suggested that vascular injury impacted the occurrence of Cis-AKI more than aging. In addition, cisplatin daily dose is associated with increased toxicity in all age groups. Daily low-dose cisplatin treatment decreases the risk of nephrotoxicity better than cyclic high-dose cisplatin treatment [28]. Our result agrees with those of a previous report and suggests that daily dose reduction of cisplatin decreases the risk of Cis-AKI. Furthermore, low BSA contributed to the prediction of Cis-AKI in patients who aged 65–74 years old. BSA was identified as a risk factor of Cis-AKI [22]. Therefore, our results agree with those of the previous study. Because low BSA results from hypoalbuminemia and aging, the evaluation of association among these factors is needed to validate our results.

In the current prediction model, the baseline serum creatinine level was not recognized as a useful predictor of Cis-AKI. Since monitoring of serum creatinine did not improve mortality and the introduction of renal replacement therapy in patients with AKI [14], our result partially agreed with the results of a previous study. Liver-type fatty acid-binding protein, urinary kidney injury molecule-1, tissue inhibitor of metalloprotease-2, and neutrophil gelatinase-associated lipocalin were identified as biomarkers of AKI [29]. Although these biomarkers might be more useful for predicting Cis-AKI than serum creatinine, methods to measure these biomarkers are limited in developing countries because of poor medical resources. To produce a prediction model accessible to many institutes and countries, we used routine clinical data [20–22] for constructing a prediction model for Cis-AKI in older patients.

Patient’s age, cisplatin dose, and serum albumin were high potential factors in the prediction model of Cis-AKI [30]. Our results agreed with the results of this previous report, and these parameters obtained from routine clinical data are valuable even in developing countries. To the best of our knowledge, no previous study has reported specific numerical values (ranges) of the parameters contributing to the prediction of Cis-AKI. The current analysis revealed that high dose of cisplatin (100–120 mg/day) and hypoalbuminemia (1.30–3.10 mg/dL) were the risk factors of Cis-AKI in all patients, whereas high dose of cisplatin (125–150 mg/day) contributed more to the occurrence of Cis-AKI than hypoalbuminemia in patients who were over 65 years old. These results might indicate the clinical insight that the dose of cisplatin needs to be adjusted in patients with hypoalbuminemia before the initiation of cisplatin based chemotherapy. In addition, older patients with hypoalbuminemia—even those with mild hypoalbuminemia—might need an adjustment of the cisplatin dose. Because the frequency of nephrotoxicity is increased in patients with low GFR, renal impairment is the cause for dose reduction of cisplatin [31,32]. However, aging and hypoalbuminemia are not defined as the criteria for dose reduction. Our prediction model can screen patients with high risk for Cis-AKI before chemotherapy. Therefore, this model might contribute to further studies in investigating the effectiveness of dose reduction in older patients with hypoalbuminemia.

The previous prediction model of Cis-AKI [30] did not use a machine learning model, such as neural networks and gradient boosting decision trees. In addition, that prediction model was not validated for older patients. Therefore, we compared the current model’s performance with previous AKI prediction models, which were constructed using neural networks and gradient boosting decision trees [16,18,19]. AUC, accuracy, precision, recall, and F-measure [33,34] were used as the main metrics for evaluating our prediction model. Although the parameters of evaluating the function of machine learning models were different among the studies, our machine learning model performed similarly with previous AKI prediction models for patients who were 65 years old and above. Prediction of drug-induced AKI was difficult in older patients because of comorbidities and polypharmacy. Therefore, detecting early-stage of AKI contributes in preventing the development end-stage kidney diseaseamong older patients. Our results suggested that the current prediction model contributes to the prevention of Cis-AKI in older patients. The number of older patients with cancer is increasing in developing countries because of the improvement in medical resources. Since the present prediction model was validated by routinely collected clinical data, this model could be used worldwide if it is validated in other countries/ethnicities.

This study had some limitations. Additional clinical data are needed to improve the performance of the prediction model. Moreover, the current prediction model was validated in a single institution. Hence, further studies are needed in other institutions to increase the validation of the current model. Although we used routine clinical data to establish the prediction model, the important factors to predict renal injury (e.g, genetics, use of nephrotoxic medications, electrolyte levels, intakes of water, volume of hydration, and magnesium supplementation) would contribute to the improvement of prediction ability of our model. Furhtermore, cisplatin-based chemotherapy was performed in the general ward, and urine volume was not measured within 6 hours in this study. Therefore, we could not evaluate renal damage by using KDIGO criteria. These factors should also be included in the future to further improve this model.

In conclusion, our prediction model for Cis-AKI performed effectively in older patients.

## Supporting information

S1 Dataset Raw data. (XLSX)
